# Accurate population proxies do not exist between 11.7 and 15 ka in North America

**DOI:** 10.1038/s41467-022-32355-4

**Published:** 2022-08-11

**Authors:** Spencer R. Pelton, Madeline E. Mackie, Robert Kelly, Todd A. Surovell

**Affiliations:** 1 Office of the Wyoming State Archaeologist, University of Wyoming, 1000 E. University Ave. Dept 3431, Laramie, WY 82071 USA; 2 Department of Sociology and Anthropology, Weber State University, 1299 Edvalson St. Dept 1208, Ogden, UT 84408 USA; 3 Department of Anthropology, University of Wyoming, 1000 E. University Ave. Dept 3431, Laramie, WY 82071 USA

**Keywords:** Archaeology, Palaeontology, Palaeoecology

**arising from** Stewart et al. *Nature Communications* 10.1038/s41467-021-21201-8 (2021)

Two recent studies in *Nature Communications* use North American radiocarbon dates as population proxies for humans and megafauna to contribute to a long-standing debate regarding the causes of extinction for at least 37 faunal genera in North America during the late Quaternary^1,2^. Broughton and Weitzel^2^ argue for mixed human and environmental causes whereas Stewart and colleagues^1^ (hereafter STE) employ radiocarbon-dated event-count modeling to conclude climate is to blame, finding no relationship between human and megafaunal populations. To some extent, our critiques pertain to both studies, but here we focus on STE because it is the most recently published and because Broughton and Weitzel^2^ employ multiple analytical techniques, a portion of which we support. We do not think the records used in STE’s analysis are robust enough to support their conclusions because they include many dates from non-archaeological contexts to depict human population and employ a faunal dataset highly degraded by taphonomic loss and biased by extensive sampling of few sites. As STE acknowledge, “we simply do not have robust records for fauna and humans for vast spans of time and space”, and we agree.

We obtained STE’s archaeological dataset from the authors to evaluate its integrity and estimate that 25.8% of the dates included in STE’s primary period of analysis between 11.7 and 15 ka (114 of 442 dates) are not derived from archaeological contexts, a problem that becomes more severe toward the early end of the dataset (Fig. 1). Of the 114 non-archaeological dates, 56 are noted specifically in the dataset’s “significance” field as “geoarchaeology”. Other non-archaeological dates are noted ambiguously as either “culture?” or “cultural?”, geoarchaeological dates (e.g., arroyo fill dates from the Agate Basin site), dates discarded by later investigations as anomalous (e.g., dates from post molds at the Paleo Crossing site), and dates from sites not widely agreed upon as archaeological (e.g., the Burning Tree Mastodon and Pendejo Cave). The remaining dataset is comprised of 328 dates from 104 archaeological sites, or 35% of the 938 dates cited as the total sample in STE (Supplementary Data 1). Except for a single date from Page-Ladson, we excluded all dates older than 14,200 cal BP using basic data hygiene, truncating the temporal span of STE’s analysis by 800 years (24% of analyzed temporal interval). An additional 600 years between 13.6 and 14.2 ka is at least 50% comprised of non-archaeological dates. A small sample of non-archaeological dates makes a big difference in the sparsely populated early tail of radiocarbon date distributions. These errors overestimate human population for at least 42% of STE’s analysis, placing people in North America prior to colonization and overestimating their abundance thereafter.

**Fig. 1:**
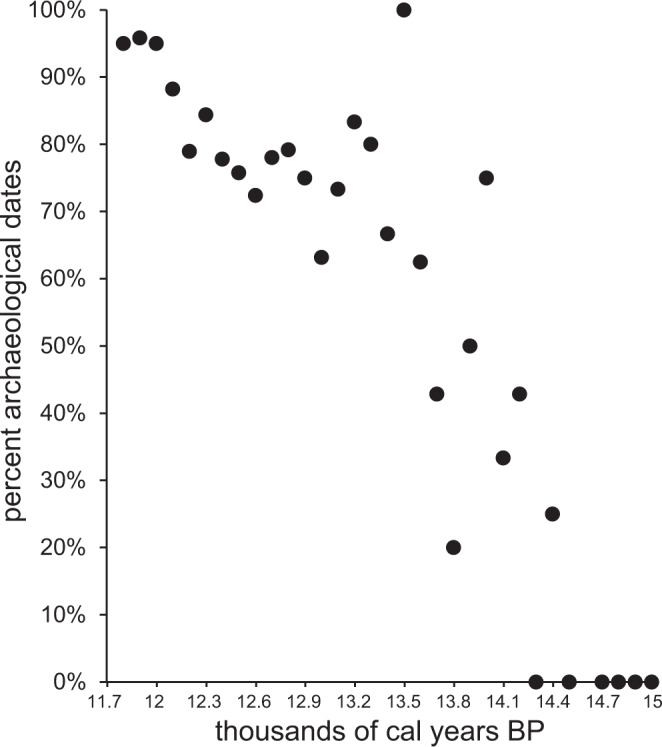
The percentage of archaeological dates associated with human occupation out of all dates in the archaeological dataset used by Stewart and colleagues’^2^ after identifying non-archaeological dates associated with geoarchaeological studies, archaeological sites not widely agreed upon as legitimate, and other questionable contexts. Dates span 11.7 to 15 ka and are binned in 100 year intervals using the median calibrated age estimate. Archaeological radiocarbon dates grow increasingly uncommon toward the past in the dataset used by Stewart and colleagues^2^, which has falsely extended the age of human occupation and overestimated human abundance for the earliest portions of the analysis.

Given the human record alone, we would expect that STE should find no relationship between faunal abundance and human population. There is currently little real human record through which a relationship can be evaluated. Even after data hygiene, archaeological evidence does not indicate continuous occupation by foragers that hunted extinct species of Pleistocene megafauna for the entire duration of STE’s analysis. As of now, that distinction belongs to the Clovis cultural complex, which at the earliest emerged ca. 13.4 ka^3^ (but perhaps as late as 13.1 ka^4^), and persisted no later than 12.7 ka, or 1000 years prior to the 11.7 ka terminal date in STE’s analysis. A more conservative correlation analysis might be constrained to the brief time during which humans certainly interacted with extinct species of Pleistocene megafauna in North America between ca. 13.4 and 12.7 ka, or 700 (21%) of the 3300 years analyzed by STE. However, concerns surrounding the quality of the faunal dataset lead us to suspect that even the use of a more conservative human dataset might return questionable results.

Systematic flaws in the faunal dataset prevent accurate population estimates due primarily to time-transgressive taphonomic loss and to some extent by sampling bias toward a few heavily-dated sites. Faunal material is lost from the geologic record of North and South America at greater rates than terrestrial sediments^5^, thus underrepresenting population proxies derived from faunal materials at progressively greater rates as one moves back in time, even after taphonomic correction procedures^6^. In a dataset primarily composed of dates derived from bone collagen, animal hide, and keratin (134 of 195 dates between 11.7–15 ka), this phenomenon should greatly impact results. Thus, there is currently no means of accurately estimating Pleistocene megafauna abundance in North America, and the attempt by STE to do so is fundamentally addressing a geologic process rather than a biological one. Given this systematic bias in the faunal dataset, we suspect STE’s positive correlation coefficients of around 0.05 to 0.20 between climate and faunal abundance is a result of autocorrelation; if one compares two phenomena that are both correlated with time, then they may also be positively correlated with each other. In this case, faunal remains decrease in abundance toward the past due to taphonomic loss just as global temperature decreases toward the last glacial maximum.

Additionally, the faunal dataset sample is biased toward a small number of heavily dated sites whose ages are more indicative of their specific formation histories than actual faunal abundance. For example, the 10 to 20 ka date range employed in STE’s ‘extended analysis’ is comprised of 103 Pleistocene faunal localities representing 259 dates, but only four western North American sites together comprise a third of the faunal dataset dates (Rancho la Brea, Bechen Cave, Rampart Cave, and Paisley Caves). Of these, the Rancho la Brea tar pit site in southern California is notable because it alone accounts for 13.5% of faunal dates between 10 and 20 ka, even after STE averaged multiple dates thought to be derived from individual animals. Dates from Rancho la Brea are clustered between tar pit deposits, a result of their individual formation histories^7^. Thus, temporal data from Rancho la Brea are fundamentally indicative of tar pit deposit formation histories rather than faunal abundance. STE are of course not accountable for the fundamentally biased sample inherent to the American Pleistocene fossil record, but they are for using it as a proxy for continuous faunal abundance.

Our critique of the faunal dataset pertains only to time-series correlation analyses that employ datasets derived from faunal remains in non-circumpolar regions. We remain optimistic that datasets resistant to extreme degrees of taphonomic loss can provide relatively accurate population proxies, even those derived from animal remains if they were recovered from circumpolar regions where animal remains are not rapidly destroyed^5^. Further, the faunal dataset employed by STE is not useless, but cannot accurately be used in time-series correlation analyses. Rather, their value lies in detecting “initial decline dates” (IDD)^8^ into extinction. Whereas radiocarbon date frequency declines toward the past are fundamentally inaccurate due to time-transgressive taphonomic loss, we remain confident that declines toward the present reflect actual decreases in the faunal populations from which they are derived because there is currently no known agent of taphonomic loss that systematically destroys progressively younger faunal remains. In this case, declines toward the present begin ca. 13 ka alongside widespread evidence for humans in North America^9^. Determining IDD has proven a compelling means of determining human influence on faunal populations^9^, including by Broughton and Weitzel^2^, who supplement a correlation analysis that we question with an analysis of IDD that we consider robust.

Accurate, continuous population records of humans and extinct Pleistocene fauna do not currently exist between 11.7 and 15 ka for North America, which leads us to question STE’s conclusion that climate change, not human population, is correlated with North American extinctions. Radiocarbon-dated event-count modeling seems like a promising means of evaluating the relatedness of time series datasets in general, but the records on which STE test the method in this study are too systematically flawed to produce accurate conclusions. We maintain that rigorous interrogation of the early Paleoindian record alongside the use of IDD in faunal datasets remains the most robust means of detecting the influence of human populations on faunal abundance during the North American late Pleistocene.

## Reporting summary

Further information on research design is available in the Nature Research Reporting Summary linked to this article.

## Supplementary information

Description of Additional Supplementary Files

Supplementary Data 1

Reporting Summary

## Data Availability

The data used to create Fig. 1 is detailed in Supplementary Data 1 of this study. These data were obtained from Stewart and colleagues to maintain consistency between our studies.
