# Supplementary material for: Accurate population proxies do not exist between 11.7 and 15 ka in North America
Source: Nat Commun. 2022 Aug 11;13:4694. doi: 10.1038/s41467-022-32355-4 (PMC9372047; doi:10.1038/s41467-022-32355-4)
Supplement: Supplementary file 1 — Description of Additional Supplementary Files [file 41467_2022_32355_MOESM1_ESM.pdf]

Description of Additional Supplementary Files

File Name: Supplementary Data 1
Description: Data table of all radiocarbon dates used in this study obtained from Stewart and colleagues noting archaeological and non-archaeological radiocarbon dates.
